# Attitudes Toward Hysterectomy in Saudi Arabian Women Undergoing Evaluation for Uterovaginal Prolapse: A Cross-Sectional Study

**DOI:** 10.7759/cureus.49967

**Published:** 2023-12-05

**Authors:** Maha Al Baalharith, Saeed AlSary, Elham Bamanie, Sameerah Al Mowallad, Joud S Makki, Sarah K Basudan, Shaden A Alotaibi, Malak E Alabdulkareem, Ahmed Abu-Zaid

**Affiliations:** 1 Department of Obstetrics and Gynecology, King Abdulaziz Medical City, Ministry of National Guard Health Affairs, Riyadh, SAU; 2 College of Medicine, King Saud Bin Abdulaziz University for Health Sciences, Riyadh, SAU; 3 College of Medicine, Alfaisal University, Riyadh, SAU

**Keywords:** saudi arabia, perceptions, attitudes, pelvic organ prolapse, uterovaginal prolapse, hysterectomy

## Abstract

Background: Hysterectomy is an effective management approach for uterovaginal prolapse. However, the decision to undergo hysterectomy is a complex matter, influenced not only by medical factors but also cultural beliefs, societal norms, and individual attitudes. In Saudi Arabia, a nation with its distinctive cultural and social norms, the understanding of women's attitudes toward hysterectomy is of utmost importance. Unfortunately, such related attitudes have not been explored.

Objective: This first-ever study aimed to investigate the attitudes toward hysterectomy among Saudi Arabian women undergoing evaluation for uterovaginal prolapse, by exploring the factors influencing their decision-making process and treatment choices, with a particular focus on the potential impact of cultural beliefs and societal norms.

Methods: A survey was conducted among 404 women referred for uterovaginal prolapse evaluation. The participants completed a self-administered questionnaire, which included demographic information, perceptions on hysterectomy's impact on well-being, and factors affecting decision-making.

Results: The mean ± standard deviation of participants was 51.07 ± 11.1 years. Most participants were currently married (n=327, 81%), were unemployed (n=309, 76.5%), and had an “excellent” self-rated general health status (n=138, 34%). Current prolapse management methods included Kegel exercises (n=103, 25.5%), pessary use (n=32, 8%), physical therapy (n=12, 3%), planned surgery (n=75, 18.5%), and no specific treatment (n=182, 45%). Overall, the study revealed diverse findings on the potential perceived impact of hysterectomy on different aspects of well-being. Notably, for pain symptoms, 152 participants (38%) reported potential improvement, while 123 participants (30%) predicted worsening, and 129 participants (32%) anticipated no change. Moreover, the study unveiled insights into the factors influencing patients' decision-making between hysterectomy and uterine-sparing procedures. Remarkably, 97 respondents (24%) considered the doctor's opinion to be "very Important," while 91 respondents (22%) rated the impact on surgical complication risk as "very important." Furthermore, the desire to preserve all healthy organs was deemed "very important" by 106 respondents (26%). The resources of information women depended on when making a decision to undergo hysterectomy varied and included a second opinion from another physician (n=68, 17%), social media (n=81, 20%), opinion from spouse/partner (21%), second opinion from female family members (n=99, 25%), and opinion from friends (n=70, 17%). Regarding preferences for decision-making, the responses varied substantially. Overall, 65 participants (16%) indicated a preference for their doctor to make the decision entirely, 81 participants (20%) preferred shared decision-making with their doctor, 89 participants (22%) wanted their doctor to make the decision after considering their input, 77 participants (19%) wished to make the final decision after discussing it with their doctor, and 93 participants (23%) expressed a preference for independently making the final decision. Lastly, correlations between women’s responses and some demographic factors were identified.

Conclusion: This pioneering study provides valuable insights into Saudi Arabian women's attitudes toward hysterectomy, emphasizing the need for patient-centered care and culturally sensitive approaches in managing uterovaginal prolapse.

## Introduction

Uterovaginal prolapse is a prevalent and distressing gynecological condition that affects a significant number of women globally [1]. It involves the descent of pelvic organs, causing various uncomfortable symptoms and diminishing the quality of life for affected individuals [2]. Among the treatment options available is hysterectomy, and it has been considered an effective and a definitive approach for the management of uterovaginal prolapse [3]. However, the decision to undergo hysterectomy is a complex and sensitive matter, influenced not only by medical factors but also cultural beliefs, societal norms, and individual attitudes [3,4].

In Saudi Arabia, a nation with its distinctive cultural and social norms, the understanding of women's attitudes toward hysterectomy is of utmost importance [5,6]. Despite the prevalence of uterovaginal prolapse and the potential benefits of hysterectomy in alleviating its symptoms, research dedicated to exploring the attitudes and perceptions of Saudi Arabian women toward this surgical procedure is notably lacking.

This study aims to address this research gap and investigate the attitudes toward hysterectomy among women undergoing evaluation for uterovaginal prolapse in Saudi Arabia. By examining the perspectives of Saudi Arabian women on hysterectomy, we can gain valuable insights into the factors influencing their decision-making process and treatment choices. Significantly, this research represents the first attempt to delve into the attitudes toward hysterectomy within the Saudi Arabian population, making it a pioneering contribution to the field.

The implications of this study go beyond mere academic inquiry. The findings hold the potential to positively impact healthcare practices and policies in Saudi Arabia, as well as other regions with similar cultural considerations [5,7]. By understanding the intricate interplay of cultural norms and individual beliefs in relation to hysterectomy, healthcare providers can offer more patient-centered and empathetic care, in addition to fostering informed decision-making and tailored treatment plans.

## Materials and methods

Between January and July of 2022, we conducted a survey among a group of women referred to our academic urogynecology practice at King Abdulaziz Medical City, Ministry of National Guard Health Affairs, Riyadh, Saudi Arabia. The participants were referred for evaluation of uterovaginal prolapse and had no previous history of hysterectomy, as confirmed through our medical records. There was no formal sample size calculation. Instead, all patients who met the inclusion criteria during the period of the study were included. The inclusion criteria included (i) confirmed diagnosis of uterovaginal prolapse, (ii) no prior history of hysterectomy, and (iii) informed consent by the patient to participate in the study.

To create the survey, we utilized an existing questionnaire on women's perceptions of hysterectomy and surgical decision-making [8]. The survey was translated into Arabic language by three physicians and one linguistic expert, and the Arabic version was used to administer the survey. Next, the survey was examined for validity by running a pilot test on seven women to warrant proper interpretation of the questions, and minor adjustments were made based on the feedback received. Notably, we have adopted a comprehensive approach to facilitate the completion of surveys for participants with limited literacy skills. We explicitly explained the survey's purpose using accessible language and offered to read questions aloud, allowing sufficient time for understanding. Emphasizing the value of their contributions, we utilized visual aids and broke down complex questions. Verbal responses were encouraged if writing proved challenging. To create a supportive environment, several trusted facilitators were present, and regular check-ins were conducted to offer assistance. Almost all patients were accompanied by educated family members who also helped patients in the process of survey completion. All in all, this approach ensured the active and meaningful participation of individuals with limited literacy skills in the survey process. Illiterate patients who failed to complete the survey reliably were excluded. 

The primary objective of the study was to gather responses from the participants with no history of hysterectomy. The survey was anonymous and self-administered during the clinic visit. The survey covered various aspects, including participants' demographics, medical history, and management strategies related to uterovaginal prolapse (see Appendix for the survey used in the study). The participants were asked to express their opinions on how they perceived a hysterectomy would impact (i.e., improve, worsen, or no change) different aspects of their well-being. They were presented with a scenario in which surgery for uterovaginal prolapse was recommended by their physician and were asked about the resources they would consult when considering surgery. A five-level scale was used to evaluate the importance of different factors in making decision regarding undergoing hysterectomy as follows: not important, mildly important, moderately important, very important, and not applicable. Lastly, the preferences for decision-making to undergo hysterectomy were examined.

For the statistical analysis, IBM SPSS Statistics for Windows (IBM Corp., Armonk, New York, United States) was used to summarize the demographic and clinical characteristics, along with the participants' responses to the survey items. Descriptive data were reported as numbers and percentages or means ± standard deviations (SD). Chi-square test was used to examine correlations between women’s responses and select demographic factors. Statistical significance was established based on two-tailed p-value less than 0.05.

The research protocol had been approved by the Institutional Review Board (IRB) at King Abdullah International Medical Research Center, Riyadh, Saudi Arabia (study number: NRC22R/066/02).

## Results

Baseline characteristics of the study participants

In this study, 404 participants were enrolled, with a mean age of 51.07 ± 11.1 years. Overall, 327 participants (81%) were currently married. The participants were geographically diverse, and their educational backgrounds and self-rated general health status varied. Employment status distribution was 10.5% full employment (n=42), 13% part-time employment (n=53), and 76.5% not working (n=309). Slightly less than half of the participants (n=205, 49%) reported their last menstrual period more than a year ago. Prolapse management methods included Kegel exercises (n=103, 25.5%), pessary use (n=32, 8%), physical therapy (n=12, 3%), planned surgery (n=75, 18.5%), or no specific treatment (n=182, 45%). Details for the baseline characteristics are tabulated in Table 1.

**Table 1:** Demographic and clinical characteristics of the participants who completed the survey. Age is presented as mean ± standard deviation, whereas all the remaining categorical variables are presented as numbers (n) and percentages (%).

Variables	Results
Age in years	51.07 ± 11.1
Currently married	327 (81%)
Level of education completed	
Primary	114 (28%)
Secondary	73 (18%)
College	111 (27.5%)
Graduate	17 (3.5%)
Illiterate	89 (22%)
Employment status	
Full employment	42 (10.5%)
Part-time employment	53 (13%)
Not working	309 (76.5%)
Self-rated general health status	
Excellent	138 (34%)
Very good	48 (12%)
Good	11 (29%)
Poor	2 (4%)
Fair	100 (25%)
Last menstrual period	
Less than one year ago	205 (51%)
More than one year ago	199 (49%)
Personal history of cancer	
None	387 (96%)
Breast	1 (0.25%)
Endometrial	2 (0.5%)
Cervical	1 (0.25%)
Other	13 (3%)
Current prolapse management	
Kegel exercise	103 (25.5%)
Pessary	32 (8%)
Planning surgery	75 (18.5%)
Physical therapy	12 (3%)
None	182 (45%)

Potential perceived impact of hysterectomy

Table 2 presents insightful data on the potential perceived impact of hysterectomy on different aspects of well-being. The data showcase the percentages of respondents who reported predicted improvement, worsening, or no change in each aspect following their potential hysterectomy procedures. Pain symptoms witnessed 152 participants (38%) reporting potential improvement, while 123 participants (30%) predicted worsening, and 129 participants (32%) anticipated no change. Regarding quality of life, 139 participants (34%) foresaw improvement, 146 participants (36%) guessed worsening, and 119 participants (29%) forecasted no change. The relationship with partners displayed mixed responses, with 111 participants (27%) reported potential improvement, 144 participants (36%) expected worsening, and 149 participants (37%) anticipated no change. This comprehensive data underscores the diverse impact of hysterectomy on physical, emotional, sexual, and social well-being, which can be valuable for healthcare providers in addressing patients' concerns and enhancing post-surgery care. Table 3 presents the p-values indicating the statistical significance of the relationships between the potential perceived impact of hysterectomy and various baseline characteristics.

**Table 2:** Potential perceived impact of hysterectomy on various aspects of physical, emotional, sexual, and social well-being among patients with uterovaginal prolapse. The data for "improve," "worsen," and "no change" responses are presented as numbers (n) and percentages (%).

Aspect	Improve	Worsen	No change
Pain symptoms	152 (38%)	123 (30%)	129 (32%)
Quality of life	139 (34%)	146 (36%)	119 (30%)
Relationship with partner	111 (27%)	144 (36%)	149 (37%)
Mood or emotional state	112 (28%)	159 (39%)	133 (33%)
Partner’s sexual satisfaction	149 (37%)	95 (24%)	160 (40%)
Sex drive	129 (32%)	137 (34%)	138 (34%)
Body weight	135 (33%)	130 (32%)	139 (35%)
Partner’s sex drive	149 (37%)	148 (37%)	107 (26%)
Body image	147 (36%)	101 (25%)	156 (39%)
Vaginal lubrication	142 (35%)	133 (33%)	129 (32%)
Sense of femininity	133 (33%)	155 (38%)	116 (29%)

**Table 3:** Correlations between the potential perceived impact of hysterectomy on different aspects of well-being and various demographic factors. Data are presented as p-values. Age was analyzed as a categorical variable (<50 and ≥50 years old). Chi-square test was used to examine correlations between women’s responses and demographic factors. Statistical significance was established based on two-tailed p-value less than 0.05.

	Pain symptoms	Quality of life	Relationship with partner	Mood or emotional state	Partner’s sexual satisfaction	Sex drive	Body weight	Partner’s sex drive	Body image	Vaginal lubrication	Sense of femininity
Age	0.228	0.078	0.455	0.198	0.048	0.521	0.547	0.753	0.059	0.165	0.121
Marital status	0.004	0.506	0.588	0.58	0.208	0.632	0.417	0.334	0.091	0.487	0.008
Level of education completed	0.209	<0.001	0.004	0.062	0.846	0.004	<0.001	<0.001	0.091	0.638	0.031
Employment status	<0.001	<0.001	0.417	0.114	0.192	0.184	0.001	<0.001	0.028	0.226	0.009
Self-rated general health status	0.021	0.006	0.193	0.094	0.075	0.164	0.46	0.041	0.1	0.304	0.002
Last menstrual period	0.591	0.001	0.443	0.278	0.139	0.489	0.726	0.751	0.804	0.248	0.013
Personal history of cancer	0.362	0.319	0.279	0.075	0.409	0.492	0.437	0.597	0.184	0.656	0.538
Current prolapse management	0.008	0.002	0.004	0.068	0.981	0.3	0.137	0.712	0.025	0.045	<0.001

Factors affecting the decision-making of hysterectomy

Table 4 provides insights into the factors influencing patients' decision-making between hysterectomy and uterine-sparing procedures for pelvic organ prolapse repair. Notably, 97 respondents (24%) considered the doctor's opinion to be "very Important," while 91 respondents (22%) rated the impact on surgical complication risk as "very important." In terms of partner-related considerations, 91 respondents (23%) emphasized the "very important" impact on their partner's opinion. Furthermore, the desire to preserve all healthy organs was deemed "very important" by 106 respondents (26%), and the influence on sexual satisfaction was also highly rated as "very important" by 100 respondents (25%). These findings provide valuable insights for healthcare professionals seeking to better understand patients' preferences and concerns when they are making decisions about pelvic organ prolapse repair options. Table 5 presents the p-values indicating the statistical significance of the relationships between the factors affecting decision-making of hysterectomy and various baseline characteristics.

**Table 4:** Importance of different factors in making decision to undergo hysterectomy for the management of uterovaginal prolapse. The data for "not important," "mildly important," "moderately important," "very important," and "not applicable" responses are presented as numbers (n) and percentages (%).

Factor	Not important	Mildly important	Moderately important	Very important	Not applicable
Doctor’s opinion	85 (21%)	100 (25%)	122 (30%)	97 (24%)	0 (0%)
Impact on surgical complication risk	81 (20%)	72 (18%)	81 (20%)	91 (22%)	79 (20%)
Impact on risk of uterine cancer	101 (25%)	108 (27%)	105 (26%)	90 (22%)	0 (0%)
Impact on pain after surgery	86 (21%)	58 (14%)	82 (20%)	76 (19%)	102 (26%)
Partner’s opinion	110 (27%)	66 (16%)	72 (18%)	91 (23%)	65 (16%)
Impact on other health problems	86 (21%)	127 (31%)	87 (22%)	104 (26%)	0 (0%)
Impact on relationship	83 (20%)	89 (22%)	65 (16%)	75 (19%)	92 (23%)
Impact on mood or emotional state	82 (20%)	104 (26%)	105 (26%)	113 (28%)	0 (0%)
Wanting to keep all healthy organs	110 (27%)	87 (22%)	101 (25%)	106 (26%)	0 (0%)
Impact on sexual satisfaction	88 (21%)	117 (29%)	99 (25%)	100 (25%)	0 (0%)
Impact on partner’s sexual satisfaction	75 (18.5%)	82 (20%)	76 (18.5%)	104 (26%)	67 (17%)
Impact on vaginal lubrication	97 (24%)	104 (26%)	113 (28%)	90 (22%)	0 (0%)
Impact on sex drive	80 (20%)	115 (28%)	93 (23%)	116 (29%)	0 (0%)

**Table 5:** Correlations between the factors affecting decision-making of hysterectomy and various baseline characteristics. Data are presented as p-values. Age was analyzed as a categorical variable (<50 and ≥50 years old). Chi-square test was used to examine correlations between women’s responses and demographic factors. Statistical significance was established based on two-tailed p-value less than 0.05.

	Doctor’s opinion	Impact on surgical complication risk	Impact on risk of uterine cancer	Impact on pain after surgery	Partner’s opinion	Impact on other health problems	Impact on relationship	Impact on mood or emotional state	Wanting to keep all healthy organs	Impact on sexual satisfaction	Impact on partner’s sexual satisfaction	Impact on vaginal lubrication	Impact on sex drive
Age	0.152	0.01	0.581	0.157	0.042	0.204	0.138	0.1	0.435	0.07	0.189	0.415	0.712
Marital Status	0.091	0.234	0.119	0.604	<0.001	0.174	0.504	0.001	0.05	0.051	0.129	0.008	0.188
Level of education completed	0.016	0.014	0.013	0.006	0.003	0.001	0.253	0.16	0.019	0.072	0.002	0.185	0.478
Employment Status	0.001	0.149	0.012	<0.001	0.36	0.024	0.006	0.606	0.822	0.213	<0.001	0.133	0.024
Self-rated general health status	0.342	0.007	0.09	0.002	<0.0001	0.002	0.135	0.116	0.106	0.024	0.463	0.134	0.618
Last menstrual period	0.75	0.038	0.85	0.025	0.077	0.207	0.122	0.343	0.637	0.359	0.468	0.028	0.488
Personal history of cancer	0.841	0.17	0.07	0.771	0.215	0.096	0.44	0.606	0.567	0.427	0.099	0.708	0.355
Sexually Active	0.002	0.15	0.826	0.137	0.375	0.029	0.003	0.186	0.094	0.172	0.259	0.617	0.991
Current prolapse management	0.024	0.711	0.067	0.035	0.179	0.001	0.005	0.018	0.198	0.001	0.967	<0.001	0.006

**Figure 1:**
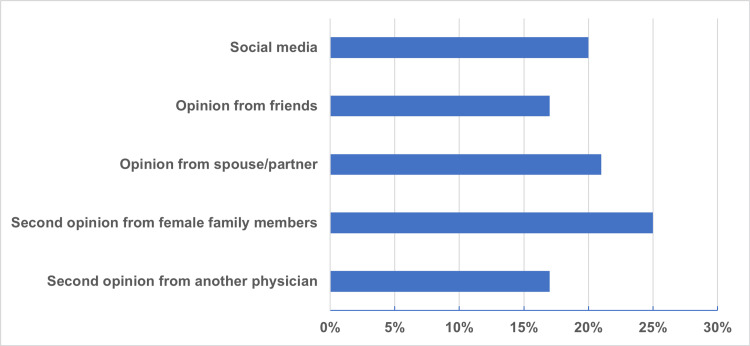
Patients' sources of information when making a decision to undergo hysterectomy for uterovaginal prolapse.

The resources of information that women depended on when making decision to undergo hysterectomy varied and included second opinion from another physician (n=68, 17%), social media (n=81, 20%), opinion from spouse/partner (21%), second opinion from female family members (n=99, 25%), and opinion from friends (n=70, 17%) (Figure 1).

**Figure 2:**
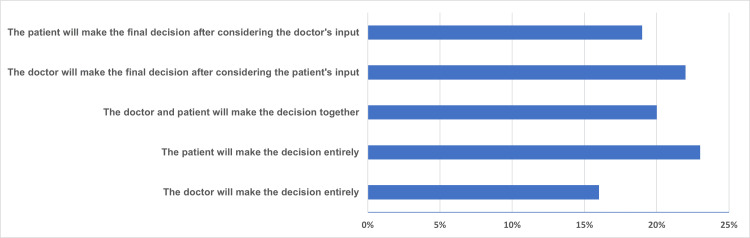
Preferences of patients for decision-making to undergo hysterectomy for uterovaginal prolapse.

Lastly, out of the 404 respondents, preferences for decision-making varied significantly (Figure 2). Overall, 65 participants (16%) indicated a preference for their doctor to make the decision entirely, while 81 participants (20%) preferred shared decision-making with their doctor. In addition, 89 participants (22%) wanted their doctor to make the decision after considering their input, and 77 participants (19%) wished to make the final decision after discussing it with their doctor. Lastly, 93 participants (23%) expressed a preference for independently making the final decision. These findings underscore the diversity of patient preferences regarding the decision-making process in medical treatment.

## Discussion

Uterovaginal prolapse is a common condition among women worldwide. According to a recent cross-sectional study of 824 participants, pelvic floor dysfunction, including prolapse, was present in 558 participants (67.7%) [9]. Another study aimed to estimate the prevalence of pelvic floor dysfunction among 2289 Saudi women attending primary healthcare centers across 13 regions of Saudi Arabia. The study found that the prevalence of uterovaginal prolapse was 23.4% (n=536) [10].

The present study included 404 participants and explored the impact of hysterectomy on well-being, revealing varied responses. Factors influencing decision-making were identified, emphasizing the importance of individualized care. These insights can inform healthcare providers in delivering personalized post-surgery care. This study's focus on the Saudi Arabian population offers an opportunity for cross-cultural comparisons, allowing for a broader understanding of attitudes toward hysterectomy across diverse societal contexts. Such comparative insights can enrich the global understanding of the psychosocial aspects influencing women's healthcare decisions, leading to more culturally sensitive and inclusive healthcare practices worldwide.

Factors influencing women's decisions on uterine preservation versus hysterectomy-based surgery for uterovaginal prolapse encompass concerns about psychological and sexual well-being, consideration of their partner's opinion, valuing the importance of keeping all healthy organs, reliance on the doctor's opinion, apprehension about surgical complication risks, and the impact of age and parity [11]. These factors were also previously highlighted in previous studies by Korbly et al. [12] and Duyar et al. [13]. Understanding these factors is crucial for healthcare providers to offer tailored care and improve patient outcomes. Open communication and providing information can help women make informed decisions about their treatment options.

Misconceptions about hysterectomy are prevalent among women, leading to challenges in understanding post-surgery expectations. Some common misconceptions include fears of mood swings, irritability, sexual dysfunction, hormonal imbalances, or depression after hysterectomy, as well as concerns about postoperative weight gain [14-17]. To address these misconceptions, patient education and informed decision-making are crucial. Healthcare providers should offer accurate information about the risks and benefits of hysterectomy and other treatment options [18-20]. Encouraging patients to ask questions and seek clarification empowers them to make decisions that align with their individual needs and preferences. A collaborative process between the patient and healthcare provider ensures that the patient can make well-informed choices regarding their health.

In Saudi Arabia, cultural and societal factors have a significant influence on women’s attitudes toward hysterectomy and uterine-sparing procedures. One potential factor is the prevalent family-centered decision-making, where families play a substantial role in medical choices. Consequently, women's attitudes toward these procedures may be influenced by the strong opinions of their families [21,22]. Religious and cultural beliefs also play a pivotal role in shaping women's attitudes. Some women may perceive hysterectomy as conflicting with their religious beliefs or cultural norms, impacting their willingness to consider the procedure [20].

The present research endeavored to shed light on a critical aspect of women's health in Saudi Arabia by examining their attitudes toward hysterectomy for uterovaginal prolapse. As the first of its kind in the Saudi Arabian context, its significance extends to improving clinical practice, healthcare policies, and the overall well-being of women. By uncovering and addressing the complex factors influencing women's decisions, this study contributes to the broader mission of delivering comprehensive and patient-centric healthcare to women, empowering them to make informed choices about their health and future.

The self-reported nature of the survey data might introduce bias due to subjective perceptions or memory recall. In addition, language and translation issues could have influenced participants' understanding of the questions. As the study utilized cross-sectional data, it only provides a snapshot of attitudes at a specific time, and longitudinal data would offer a more comprehensive perspective. Moreover, reporting data from a single-center institute limited our ability to generalize the outcomes to Saudi Arabia. Moreover, the exclusion of women with a history of hysterectomy might have overlooked important insights from previously hysterectomized women. An additional shortcoming includes the lack of evaluating the functional severity of uterovaginal prolapse, which could have influenced the perceived attitudes toward undergoing hysterectomy. Lastly, the present study did not explore cross-cultural differences or perform a comparative analysis between women opting for hysterectomy and uterine-sparing procedures. Despite these limitations, the study contributes valuable insights into the factors influencing women's decision-making process in the context of hysterectomy and can inform healthcare practices in Saudi Arabia and similar cultural settings.

## Conclusions

This study sheds light on the attitudes toward hysterectomy among Saudi Arabian women undergoing evaluation for uterovaginal prolapse. The findings offer valuable insights into the factors that influence their decision-making process and treatment choices, with cultural beliefs and societal norms playing a significant role. The research reveals diverse perspectives on the impact of hysterectomy on various aspects of well-being, highlighting the complexity of this surgical decision.

## Appendices

Survey used in the study 

**Table 6:** Survey used in the study.

Section 1. The demographic and clinical characteristics of participants completing the survey. (choose only one answer)							
Age in years							
Are you currently married							
Yes							
No							
Level of education completed							
Primary							
Secondary							
College							
Graduate							
Illiterate							
Employment status							
Full employment							
Part-time employment							
Not working							
Self-rated general health status							
Excellent							
Very good							
Good							
Poor							
Fair							
Last menstrual period							
Less than one year ago							
More than one year ago							
Personal history of cancer							
None							
Breast							
Endometrial							
Cervical							
Other							
Current prolapse management							
Kegel exercise							
Pessary							
Planning surgery							
Physical therapy							
None							
Section 2. The potential perceived impact of hysterectomy on various aspects of physical, emotional, sexual, and social well-being among patients undergoing hysterectomy for uterovaginal prolapse. (choose only one answer)							
Aspect	Improve		Worsen			No change	
Pain symptoms							
Quality of life							
Relationship with partner							
Mood or emotional state							
Partner’s sexual satisfaction							
Sex drive							
Body weight							
Non-gynecologic health problems							
Partner’s sex drive							
Body image							
Vaginal lubrication							
Sense of femininity							
Section 3. Importance of different factors in making decision to undergo hysterectomy for management of uterovaginal prolapse. (choose only one answer)							
Factor	Not important	Mildly important		Moderately important	Very important		Not applicable
Doctor’s opinion							
Impact on surgical complication risk							
Impact on risk of uterine cancer							
Impact on pain after surgery							
Partner’s opinion							
Impact on other health problems							
Impact on relationship							
Impact on mood or emotional state							
Wanting to keep all healthy organs							
Impact on sexual satisfaction							
Impact on partner’s sexual satisfaction							
Impact on vaginal lubrication							
Impact on sex drive							
Section 4. The preferences for decision-making to undergo hysterectomy for management of uterovaginal prolapse. (choose only one answer)							
I want the doctor to make the decision entirely							
I want to make the decision myself entirely							
I want to make the decision together with the doctor							
I want the doctor to make the final decision after considering my input							
I want to make the final decision myself after discussing it with the doctor							

## References

[1] Wang B, Chen Y, Zhu X (2022). Global burden and trends of pelvic organ prolapse associated with aging women: An observational trend study from 1990 to 2019. Front Public Health.

[2] Weintraub AY, Glinter H, Marcus-Braun N (2020). Narrative review of the epidemiology, diagnosis and pathophysiology of pelvic organ prolapse. Int Braz J Urol.

[3] Janda M, Armfield NR, Page K (2018). Factors influencing women's decision making in hysterectomy. Patient Educ Couns.

[4] Jeppson PC, Sung VW (2014). Hysterectomy for pelvic organ prolapse: indications and techniques. Clin Obstet Gynecol.

[5] El-Islam MF (2005). Some cultural aspects of the Arab patient-doctor relationship. Int Psychiatry.

[6] Kronfol NM (2012). Access and barriers to health care delivery in Arab countries: a review. East Mediterr Health J.

[7] Tayeb HO, Tekian A, Baig M, Koenig HG, Lingard L (2023). The role of religious culture in medical professionalism in a Muslim Arab society. Perspect Med Educ.

[8] Frick AC, Barber MD, Paraiso MF, Ridgeway B, Jelovsek JE, Walters MD (2013). Attitudes toward hysterectomy in women undergoing evaluation for uterovaginal prolapse. Female Pelvic Med Reconstr Surg.

[9] Malaekah H, Al Medbel HS, Al Mowallad S, Al Asiri Z, Albadrani A, Abdullah H (2022). Prevalence of pelvic floor dysfunction in women in Riyadh, Kingdom of Saudi Arabia: A cross-sectional study. Womens Health (Lond).

[10] Al-Badr A, Saleem Z, Kaddour O (2022). Prevalence of pelvic floor dysfunction: a Saudi national survey. BMC Womens Health.

[11] Ramage K, Ducey A, Scime NV, Knox E, Brennand EA (2023). Factors affecting women's decision between uterine-preserving versus hysterectomy-based surgery for pelvic organ prolapse. Womens Health (Lond).

[12] Korbly NB, Kassis NC, Good MM (2013). Patient preferences for uterine preservation and hysterectomy in women with pelvic organ prolapse. Am J Obstet Gynecol.

[13] Duyar S, Tsai S, Milad MP, Chaudhari A (2023). Attitudes and beliefs about hysterectomy in patients with uterine fibroids. J Minim Invasive Gynecol.

[14] McCracken G, Lefebvre GG (2007). Vaginal hysterectomy: dispelling the myths. J Obstet Gynaecol Can.

[15] Raza N, Waqas A, Jamal M (2015). Post-operative anxiety, depression and psychiatric support in patients undergoing hysterectomy: A cross sectional survey. J Pak Med Assoc.

[16] PA RM, CR JB (1963). Misconceptions concerning the psychological effects of hysterectomy. Am J Obstet Gynecol.

[17] Bossick AS, Sangha R, Olden H, Alexander GL, Wegienka G (2018). Identifying what matters to hysterectomy patients: postsurgery perceptions, beliefs, and experiences. J Patient Cent Res Rev.

[18] Haslett S (1985). Health education. Hysterectomy counselling. Nurs Mirror.

[19] Neefus MS, Taylor ME (1982). Educational needs of hysterectomy patients. Patient Couns Health Educ.

[20] Dulaney PE, Crawford VC, Turner G (1990). A comprehensive education and support program for women experiencing hysterectomies. J Obstet Gynecol Neonatal Nurs.

[21] Alfahmi MZ (2022). Patients' preference approach to overcome the moral implications of family-centred decisions in Saudi medical settings. BMC Med Ethics.

[22] Muaygil R (2018). Her uterus, her medical decision? Dismantling spousal consent for medically indicated hysterectomies in Saudi Arabia. Camb Q Healthc Ethics.

